# Determinants influencing municipal decision-makers in Germany regarding community health promotion: Lisa Paulsen

**DOI:** 10.1093/eurpub/ckac130.221

**Published:** 2022-10-25

**Authors:** L Paulsen, L Benz, C Müller, B Wallmann-Sperlich, J Bucksch

**Affiliations:** 1 Prevention and Health Promotion, Heidelberg University of Education, Heidelberg, Germany; 2 Institute for Sport Sciences, University of Würzburg, Würzburg, Germany

## Abstract

**Background:**

Community health promotion should be based on theoretical behavioural and environmental approaches. Especially changes in the physical environment require administrative and political decisions. This study aims to identify determinants that influence decision-makers from local politics and administrations on decisions so that health promotion can be placed on the agenda of communities as a prerequisite of intervention implementation.

**Methods:**

We used the methods of qualitative guided interviews and a quantitative survey. First, decision-makers from local politics and administrations in both urban and rural areas in Germany were interviewed in the period from July to November 2020. The interviews were analysed using qualitative content analysis according to Kuckartz and MAXQDA. Second, a nationwide online survey was conducted using Limesurvey. We used descriptive analyses. In both surveys, decision-makers reported the determinants for decision-making processes and their decision-making behaviour.

**Results:**

22 interviews were conducted (women n = 7), and 415 participants (women n = 118) responded to the online survey. The decision-making behaviour of local decision-makers can be differentiated on different levels, following socio-ecological models: individual, socio-cultural, institutional, municipal and political. Each of these levels comprises a multitude of determinants that are essential for successful persuasion toward community health promotion. At the individual level, we identified determinants like attitudes, outcome expectations or emotions towards a topic.

**Conclusions:**

The identification and understanding of determinants for local decision-making are essential for a tailored and theory-based intervention approach to place health promotion on the agenda of communities and to implement interventions. Further research is needed to replicate the importance of potential determinants and to develop effective intervention methods and techniques.

**Key messages:**

- Regarding to a theory-based implementation of community health, determinants of local decision-makers should be taken into account.
- The determinants are nested on different levels (e.g. individual, socio-cultural, institutional) of a socio-ecological approach.

